# A possible role of low regulatory T cells in anti-acetylcholine receptor antibody positive myasthenia gravis after bone marrow transplantation

**DOI:** 10.1186/s12883-017-0881-7

**Published:** 2017-05-15

**Authors:** Masahiko Fukatsu, Takenobu Murakami, Hiroshi Ohkawara, Shunichi Saito, Kazuhiko Ikeda, Suguru Kadowaki, Itaru Sasaki, Mari Segawa, Tomoko Soeda, Akihiko Hoshi, Hiroshi Takahashi, Akiko Shichishima-Nakamura, Kazuei Ogawa, Yoshihiro Sugiura, Hitoshi Ohto, Yasuchika Takeishi, Takayuki Ikezoe, Yoshikazu Ugawa

**Affiliations:** 1 Department of Neurology, Fukushima Medical University, 1 Hikariga-oka, Fukushima, Fukushima 960-1295 Japan; 2 Department of Hematology, Fukushima Medical University, Fukushima, Japan; 3 Department of Blood Transfusion and Transplantation Immunology, Fukushima Medical University, Fukushima, Japan; 4 Department of Cardiovascular Medicine, Fukushima Medical University, Fukushima, Japan; 5 Fukushima Global Medical Science Center, Advanced Clinical Research Center, Fukushima Medical University, Fukushima, Japan

**Keywords:** Myasthenia gravis, Hematopoietic cell transplantation, Graft-versus-host disease, Regulatory T cells, Rituximab, Anti-acetylcholine receptor antibody

## Abstract

**Background:**

Chronic graft-versus-host disease (GVHD) appears several months following allogenic hematopoietic stem cell transplantation (HSCT) and is clinically analogous to autoimmune disorder. Polymyositis is a common neuromuscular disorder in chronic GVHD, but myasthenia gravis (MG) is extremely rare. Hence, its pathophysiology and treatment have not been elucidated.

**Case presentation:**

A 63-year-old man with a history of chronic GVHD presented with ptosis, dropped head, and dyspnea on exertion, which had worsened over the previous several months. He showed progressive decrement of compound muscle action potential in the deltoid muscle evoked by 3-Hz repetitive nerve stimulation, a positive edrophonium test, and elevated levels of serum anti-acetylcholine receptor antibodies, which suggested a diagnosis of generalized MG. No thymoma was found. Flow cytometric analysis revealed a remarkable depletion of peripheral Tregs (CD4^+^CD25^high^FOXP3^+^ cells, 0.24% of the total lymphocytes). Administration of prednisolone and tacrolimus was insufficient to alleviate his symptoms; however, the use of rituximab successfully improved his condition.

**Conclusions:**

Myasthenic symptoms appeared in the process of tapering prednisolone for the treatment of chronic GVHD, supporting the diagnosis of MG associated with chronic GVHD. The present case proposes a possibility that reduction of Tregs might contribute to the pathogenesis of MG underlying chronic GVHD. Immunotherapy with rituximab is beneficial for treatment of refractory MG and GVHD.

## Background

Myasthenia gravis (MG) is a neuromuscular disorder characterized by muscle weakness and pathological fatigability of skeletal muscles. The pathophysiology of MG is defined as the production of autoantibodies blocking acetylcholine receptors at the neuromuscular junction [1]. Chronic graft-versus-host disease (GVHD) is mediated by the reactivation of donor T cells against recipient tissues and appears several months after allogenic hematopoietic stem cell transplantation (HSCT) in most cases [2]. The major organs involved in GVHD include the skin, gastrointestinal tract, and liver, whereas chronic GVHD features a wide variety of autoimmune disorders, including Sjögren syndrome, scleroderma, bronchiolitis obliterans, and immune cytopenias [3]. Chronic GVHD also affects neuromuscular system. In fact, polymyositis is a common neuromuscular disease present in chronic GVHD; however, MG is extremely rare. Although the 2015 National Institutes of Health Consensus Conference categorized MG under “other features or unclassified entities” of the signs and symptoms for diagnosis and staging of chronic GVHD, there have been a limited number of MG cases following allogeneic HSCT [4], and its pathophysiology and treatment approach have not yet been well established.

We present a case of chronic GVHD developing generalized MG that was successfully treated with advanced immunotherapy. The current case revealed a marked reduction of regulatory T cells (Tregs), suggesting the possible pathogenesis of MG in patients with chronic GVHD.

## Case presentation

A 63-year-old man without familial history of MG was diagnosed with secondary acute myeloid leukemia that originated from myelodysplastic/myeloproliferative neoplasms, unclassifiable 2 years prior to the current presentation. He was then treated with intensive chemotherapy, and underwent allogeneic HSCT from a human leukocyte antigen (HLA)-matched unrelated donor in the following year. Prophylaxis against GVHD consisted of tacrolimus and short-term methotrexate. He achieved remission of acute GVHD, and tacrolimus was discontinued on day 86. He then developed mild chronic GVHD of the skin and liver at 7 and 12 months after the transplantation, respectively. Fourteen months after the transplantation, he was admitted to our hospital due to progressive bilateral pleural effusion, which was attributed to pleuritis related to chronic GVHD, and was successfully treated with intravenous methylprednisolone pulse therapy (mPSL) (1 g during the course of 3 days) followed by oral prednisolone (1 mg/kg/day). During the process of tapering oral prednisolone to 7.5 mg/day (20 months after the transplantation), the patient began to complain of bilateral ptosis, dropped head, and dyspnea on exertion, which continued to worsen, and he was admitted to our hospital.

On examination, his general condition was normal, except for the presence of sinus tachycardia (106/min). He had a moon face appearance as well as increased pigmentation and sclerotic changes on the skin. He was alert and had normal cognitive function. The patient had ptosis, dropped head, and mild bilateral weakness involving the craniocervical muscles and deltoid muscles (grade 4 as measured by the Manual Muscle Strength Testing) with fatigability. However, no bulbar symptoms were noted.

Blood sample tests showed an elevated anti-acetylcholine receptor (AChR) antibody (14.0 nmol/L), but negative anti-muscle specific kinase (anti-MuSK) antibody. His HLA genotype consisted of A*24:02-B*52:01-C*12:02-DRB1*12:01 and A*24:02-B*52:01-C*12:02-DRB1*15:02. Although the arterial blood carbon dioxide concentration was within the normal range, a pulmonary function test revealed a substantial reduction of vital capacity (VC) at 1.69 L (45.4% predicted). Polymerase chain reaction analysis of short tandem repeat sequences for peripheral blood showed 100% donor chimerism. Chest computed tomography suggested mild emphysematous changes in the lung field, but thymoma was not found. Intravenous injection of edrophonium remarkably improved the patient’s ptosis and dropped head. An electrophysiological study showed 20.0% area decrement of compound muscle action potentials (CMAP) from the right deltoid muscle by 3-Hz repetitive axillary nerve stimulation. These findings confirmed a diagnosis of generalized MG with Myasthenia Gravis Foundation of America clinical classification IIb, quantitative MG score 14 out of 36, and MG-ADL score 8 out of 24.

**Fig. 1:**
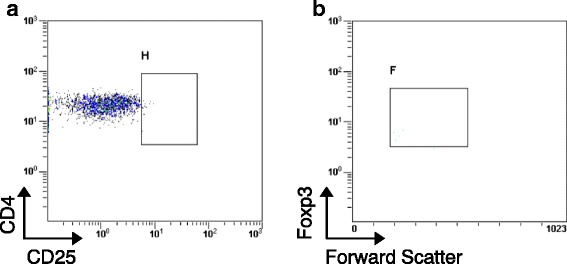
Decreased CD4^+^CD25^high^FOXP3^+^ cells (Tregs) in peripheral blood mononuclear cells from the patient. **a** Reduced proportion of CD25^high^ cells in gated CD4^+^ fraction in peripheral blood mononuclear cells (gate H). **b** Most CD4^+^CD25^high^ cells in gate H (>90%) were positive for Foxp3 (gate F)

Flow cytometric analyses were performed by using the patient’s peripheral blood because depletion of Tregs after HSCT has been reported to be valuable in predicting chronic GVHD severity [5–7]. The Treg population in the peripheral blood, defined as CD4^+^CD25^high^FOXP3^+^ cells, was 0.24% of the total lymphocytes (Fig. 1). This proportion was lower than that of our post-HSCT patients (mean ± SD 0.51 ± 0.15%) and of healthy subjects (0.94 ± 0.19%) [5].

**Fig. 2:**
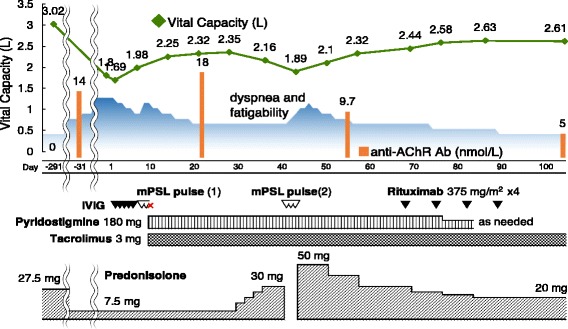
Clinical courses of the present case. VC (in L) and anti-AChR antibody titer (in nmol/L) are shown in a line chart and a bar graph, respectively. Intravenous immunoglobulin was initially administered (0.4 g/kg for 5 days) with only moderate response. Two courses of mPSL pulse treatment (1 g for 2 to 3 days) rather exacerbated MG symptoms. Meanwhile, he was started on pyridostigmine 180 mg/day and tacrolimus 3 mg/day. Finally, he was administered four courses of weekly rituximab 375 mg/m^2^, and his fatigability and weakness continued to improve steadily without pyridostigmine

Considering progression to myasthenic crisis, intravenous immunoglobulin was initially administered (0.4 g/kg for 5 days) and the patient showed mild responses for his ptosis and dropped head, but his fatigability and dyspnea on exertion remained unchanged. Subsequent mPSL pulse treatment (1 g for 2 days) exacerbated MG symptoms. We started pyridostigmine 180 mg/day with tacrolimus 3 mg/day, and consequently, his fatigability gradually improved and VC increased to 2.35 L (63.1% predicted) on the 28th hospital day. Follow-up electrophysiological study showed disappearance of significant CMAP decrement (4.5% area decrease). However, these improvements were insufficient for his daily activity. Follow-up anti-AChR antibody was still elevated to 18.0 nmol/L on the 22nd hospital day. The patient was treated with an increased oral prednisolone dose of 50 mg/day and intravenous mPSL pulse (1 g for 3 days), and anti-AChR antibody was decreased to 9.7 nmol/L on the 55th hospital day. Finally, he was administered four courses of weekly rituximab 375 mg/m^2^, and his fatigability and weakness continued to improve steadily without pyridostigmine. He was discharged on the 92nd hospital day, receiving tacrolimus 3 mg/day and prednisolone 20 mg/day (Fig. 2). Five months after discharge, minimal manifestations remained, with a VC of 2.95 L (79.2% predicted), and he continued to take oral prednisolone at a dose of 10 mg/day and tacrolimus at 3 mg/day. Titers of anti-AChR antibody were steadily reduced (4.6, 3.7, and 3.2 nmol/L on 3, 6, and 9 months after discharge).

## Discussion and conclusions

MG is a very rare complication following HSCT, with an occurrence of less than 1%. Myasthenic symptoms typically develop between 22 and 60 months after transplantation [8] and most reported cases are associated with the existence of other symptoms of chronic GVHD, as MG is rarely the sole manifestation [9]. Myasthenic symptoms appear after the discontinuation or tapering of immunosuppressive agents [10], as in the present case. Patients with aplastic anemia as a background would have an increased risk of developing MG after transplantation [11]. Patients with specific HLAs (HLA Cw1, Cw7 or DR2) and a family history of MG are also at an increased risk of developing MG after HSCT [12, 13]. However, none of these risk factors was identified in the present case.

Most patients with MG associated with chronic GVHD test positive for anti-AChR antibodies. Approximately 20% of patients with chronic GVHD show positive anti-AChR antibody without myasthenic symptoms, indicating the existence of higher rates of subclinical MG [14]. In the present case, an elevated titer of anti-AChR antibody supported the diagnosis of MG, and its titer decreased in parallel with the improvement of myasthenic symptoms after immunotherapies. A few reported patients with MG after transplantation revealed other antibodies toward non-AChR components of the postsynaptic muscle endplate, e.g. anti-MuSK and anti-striated muscle antibodies [15, 16]. These findings suggest that pathogenic autoantibodies toward the neuromuscular junctions may be produced when discontinuing or tapering immunosuppressive treatments after transplantation.

A unique point in the present case was lower population of Tregs in the peripheral blood. Tregs are a subset of T lymphocytes that modulate the immune system and maintain tolerance to self-antigens [17, 18]. The frequency of Tregs in the peripheral blood has been reported to negatively correlate with GVHD severity [6]. Absence of Tregs coupled with donor-derived T cells leads to development of GVHD [19]. The number of Tregs in the peripheral blood decreases in untreated MG and is normalized by immunotherapy [20]. A functional impairment of thymic Tregs has been reported in the thymus of patients with MG and may play a role in triggering the autoimmune process [21]. Recent studies have reported Treg dysfunction as well as its downstream signal transducer and activator of transcription pathway is associated with immunopathology of MG, and a Treg-based immunotherapeutic approach has been suggested for experimental models of MG [22–24]. We suspect that suppression of Tregs in the present case may have partly contributed to the pathogenesis of MG as a manifestation of chronic GVHD.

Recently, several reports have shown that rituximab, a monoclonal antibody against B cell surface antigen CD20, could be an effective treatment for patients with refractory MG [25, 26]. Rituximab therapy also reduces the incidence of GVHD following allogeneic HSCT and exhibits a beneficial effect on steroid-refractory GVHD [27]. Although rituximab itself directly interacts with B cells, B cell depletion subsequently leads to expansion of Tregs and suppression of autoreactive T cells [28]. Additionally, treatment with rituximab was shown to be successful in two cases of MG associated with chronic GVHD [27, 29]. This evidence supports the utility of rituximab for refractory GVHD and MG against conventional immunotherapies in the present case.

In conclusion, we reported a rare case of chronic GVHD developing systemic MG after allogenic HSCT. Appearance of myasthenic symptoms during the process of tapering prednisolone for the treatment of chronic GVHD is a key feature of MG associated with chronic GVHD. We hypothesize that the reduced number of Tregs might play a possible role in the pathogenesis of MG and chronic GVHD. Immunotherapy with rituximab led to improvements of the patient’s myasthenic symptoms, supporting the utility of rituximab for refractory MG and GVHD.

## Abbreviations

AChR: Anti-acetylcholine receptor
anti-MuSK: Anti-muscle specific kinase
CMAP: Compound muscle action potentials
GVHD: Graft-versus-host disease
HLA: Human leukocyte antigen
HSCT: Hematopoietic stem cell transplantation
MG: Myasthenia gravis
mPSL: Methylprednisolone
Tregs: Regulatory T cells
VC: Vital capacity
